# Imaging-Based Reporter Systems to Define CVB-Induced Membrane Remodeling in Living Cells

**DOI:** 10.3390/v12101074

**Published:** 2020-09-25

**Authors:** Nicholas J. Lennemann, Azia S. Evans, Carolyn B. Coyne

**Affiliations:** 1 Department of Pediatrics, University of Pittsburgh School of Medicine, Pittsburgh, PA 15224, USA; aziaevans@pitt.edu (A.S.E.); coynec2@pitt.edu (C.B.C.); 2 Center for Microbial Pathogenesis, UPMC Children’s Hospital of Pittsburgh, Pittsburgh, PA 15224, USA; 3 Richard K. Mellon Institute for Pediatric Research, UPMC Children’s Hospital of Pittsburgh, Pittsburgh, PA 15224, USA

**Keywords:** enterovirus, coxsackievirus, positive strand RNA virus, reporter, live-cell imaging

## Abstract

Enteroviruses manipulate host membranes to form replication organelles, which concentrate viral and host factors to allow for efficient replication. However, this process has not been well-studied in living cells throughout the course of infection. To define the dynamic process of enterovirus membrane remodeling of major secretory pathway organelles, we have developed plasmid-based reporter systems that utilize viral protease-dependent release of a nuclear-localized fluorescent protein from the endoplasmic reticulum (ER) membrane during infection, while retaining organelle-specific fluorescent protein markers such as the ER and Golgi. This system thus allows for the monitoring of organelle-specific changes induced by infection in real-time. Using long-term time-lapse imaging of living cells infected with coxsackievirus B3 (CVB), we detected reporter translocation to the nucleus beginning ~4 h post-infection, which correlated with a loss of Golgi integrity and a collapse of the peripheral ER. Lastly, we applied our system to study the effects of a calcium channel inhibitor, 2APB, on virus-induced manipulation of host membranes. We found that 2APB treatment had no effect on the kinetics of infection or the percentage of infected cells. However, we observed aberrant ER structures in CVB-infected cells treated with 2APB and a significant decrease in viral-dependent cell lysis, which corresponded with a decrease in extracellular virus titers. Thus, our system provides a tractable platform to monitor the effects of inhibitors, gene silencing, and/or gene editing on viral manipulation of host membranes, which can help determine the mechanism of action for antivirals.

## 1. Introduction

Positive-strand RNA viruses represent a large group of viruses that are responsible for the development of severe disease manifestations worldwide. Enteroviruses, including coxsackievirus B3 (CVB) and enterovirus 71 (EV71), are small, non-enveloped, positive-strand RNA viruses. Infection by these viruses can lead to the development of severe disease, including acute flaccid paralysis, meningitis, and encephalitis [1,2,3,4,5,6]. Currently, there are no antivirals and vaccines are only available for enterovirus 71 and poliovirus. Thus, a better understanding of the interactions of these viruses with the host cell can aid in the development of anti-enterovirus small molecule therapeutics.

All positive-strand RNA viruses manipulate host cell membranes to form membranous structures, termed replication organelles (ROs), that concentrate viral and host factors to allow for efficient genome replication [7,8,9,10]. Enteroviruses induce extensive remodeling of the host secretory pathway during infection, which results in the accumulation of large clusters of vesicular structures in the cytoplasm that harbor viral replication proteins [11,12,13,14]. Previous studies have shown that at early stages of enterovirus infection, ROs are observed in contact with the endoplasmic reticulum (ER) membrane [10,15]. As infection progresses, the accumulation of viral nonstructural proteins 3A and 2B leads to an inhibition of secretory trafficking, resulting in the dispersal of the Golgi complex [14,16,17,18,19,20]. The infection eventually leads to the release of progeny virion through disruption of the plasma membrane or non-lytic release [21]. While these events have been well-studied using traditional imaging techniques on fixed samples, the single-cell kinetics have not been well defined.

Imaging of virus-infected cells typically involves taking still images of fluorescently labeled fixed samples. While these methods are widely used and highly practical, there are a number of limitations. The choice of fixation and permeabilization reagents can have a significant impact on the outcome of immunofluorescence staining [22,23,24]. Additionally, imaging of fixed samples only allows for ‘snapshots’ of different cells at various time points, but infection is a dynamic process. Thus, a large sample size is needed to accurately confirm observed phenotypes. To overcome these limitations, live-cell imaging has been used in combination with recombinant viruses encoding fluorescent reporter proteins, such as GFP. These recombinant viruses are highly beneficial for many assays, including high-throughput screens. However, the insertion of a fluorescent protein open reading frame in small positive-strand RNA viruses, including enteroviruses and flaviviruses, can dramatically attenuate viral progeny [14,25,26], thereby limiting the scope of assays for which these viruses can provide accurate information. Previous reports have overcome these limitations by developing plasmid-based reporters to detect hepaci- and flavivirus infections [27,28,29]. These reporters rely on viral protease-dependent cleavage to allow for the translocation of a fluorescent protein to the nucleus from the mitochondria [27] or the ER [28,29]. However, these reporters only detect virus infection and do not allow for the characterization of virus-induced remodeling of intracellular host cell structures without the introduction of multiple fluorescent protein marker expression vectors. Thus, we sought to develop plasmid-based multipartite reporters for live-cell imaging that allow for the visualization of host cell organelles and enterovirus infection.

In this report, we describe the characterization of enterovirus reporter constructs that allow for the visualization of the ER and Golgi complex throughout infection. Using long-term time-lapse imaging over the complete course of infection, we define the real-time remodeling of host secretory pathway organelles during CVB infection.

## 2. Materials and Methods

### 2.1. Cell Culture and Viruses

HeLa cells were cultured in modified Eagle’s medium supplemented with 5% fetal bovine serum, 1× non-essential amino acids, and 1% penicillin/streptomycin. U2OS osteosarcoma cells were cultured in Dulbecco’s modified Eagle’s medium supplemented with 10% fetal bovine serum and 1% penicillin/streptomycin. U2OS cells were transfected using XtremeGeneHP (Roche, Mannheim, Germany), and selected and maintained in 15 µg/mL and 2.5 µg/mL blasticidin S HCl (Research Products International Corp, Mount Prospect, IL, USA), respectively. Heterogeneous populations of stable cells were propagated and used for experiments. All enteroviruses were propagated in HeLa cells and stocks were prepared and titrated by plaque assay, as previously described [30].

### 2.2. Plasmid Construction

Plasmids expressing myc-tagged wild-type and catalytically inactivated CVB 3C^pro^ have previously been described [31,32]. All primer sequences for cloning are listed in Supplementary Materials Table S1. RepER: A PCR fragment corresponding to amino acids 280–368 of the poliovirus receptor, which includes the type I transmembrane region, was amplified with flanking KpnI and EcoRI restriction sites from cDNA and cloned into pcDNA3.1-V5-His TOPO TA vector. The ER-localized mCherry-KDEL (a gift from Gia Voeltz, University of Colorado), which includes the 5′ BiP signal sequence and a 3′ flavivirus octapeptide host signal peptidase cleavage sequence (LVNSLVTA), was amplified and inserted upstream of the PVR fragment at HindIII and KpnI sites. Next, an enterovirus 3C^pro^ cleavage sequence (LEAEFQ↓GPPK) flanked by EcoRI and BamHI sites was inserted with an amplified GFP with the c-myc nuclear localization sequence (PAAKRVKLD) flanked by BamHI and XhoI sites into the vector. Lastly, the neomycin resistance gene was replaced with a blasticidin resistance gene using Gibson assembly (NEB, Ipswich, MA, USA). To generate the RepER mutant that is unable to be cleaved by 3C^pro^, the protease recognition-GFP-NLS fragment was amplified and ligated into RepER at EcoRI-XbaI sites. RepOr: the GFP-NLS sequence in RepER was replaced with an eBFP2-NLS sequence, EBFP2-Nucleus-7 was a gift from Michael Davidson (Addgene plasmid # 55249). A fluorescent Golgi marker, mEmerald-Golgi-7 (a gift from Michael Davidson, Addgene plasmid # 54108), flanked by HindIII and EcoRI was produced by PCR. Next, the EMCV IRES sequence flanked by EcoRI and NotI was amplified. The new RepER cassette containing eBFP2-NLS flanked by NotI and XbaI was amplified and ligated with mEmerald-Golgi-7 and EMCV-IRES amplicons into pcDNA3.1 RepER plasmid at the HindIII and XhoI.

### 2.3. Antibodies and Reagents

Rabbit polyclonal antibodies against c-myc (A-14) and GFP (FL) were purchased from Santa Cruz Biotechnology. Rabbit polyclonal antibodies against GAPDH were purchased from Proteintech. Mouse monoclonal antibodies against vinculin, clone hVIN-1, were purchased from Sigma Aldrich (St. Louis, MO, USA). Mouse monoclonal antibodies against enterovirus VP1 (NCL-entero) were purchased from Novocastra (Buffalo Grove, IL, USA). XtremeGene HP plasmid transfection reagent was purchased from Sigma Aldrich. Brefeldin A (Invitrogen, Carlsbad, CA, USA) was dissolved in DMSO and used at a final concentration of 5 µg/mL. Also, 2-aminoethoxydiphenylborane (2APB, Sigma Aldrich) was diluted in DMSO and used at a final concentration of 100 µM.

### 2.4. Virus Infections

CVB growth kinetics were determined using U2OS cells transfected with the indicated plasmids. U2OS cells were bound with CVB (100 PFU/cell) for 1 h at 4 °C, washed ×3 in PBS, and placed at 37 °C. At the indicated time, samples were collected from infected cells and used for plaque assays to determine the titer of the extracellular virus. Inhibition of infection using 2APB was performed in U2OS cells. Cells were treated with 2APB (100 µM) for 1 h at 37 °C, CVB (50 PFU/cell) was absorbed for 1 h in the presence of 2APB at room temperature with gentle rocking, washed three times, and replaced with growth media containing 2APB for 10 h. Supernatants were collected to determine the extracellular virus titer by plaque assay.

### 2.5. Immunoblots

U2OS cells or HeLa cells were transfected with the indicated plasmids using XtremeGeneHP (Roche). When specified, HeLa and U2OS cells were infected with the indicated virus for 7 h. Cell lysates were prepared on ice in cold RIPA buffer (Millipore, 50 mM Tris-HCl, pH7.4, 1% NP-40, 0.25% sodium deoxycholate, 150 mM NaCl, 1 mM EDTA) supplemented with 1× Pierce protease inhibitor cocktail (AEBSF, aprotinin, bestatin, E64, leupeptin, pepstatin A). Lysates were clarified by centrifugation at 13,000× *g* for 15 min at 4 °C. Lysates (10–30 µg) were separated on 4–20% TGX pre-cast gels (Bio-Rad Laboratories, Hercules, CA, USA) and transferred to nitrocellulose membranes. Membranes were blocked in PBS + 10% nonfat milk for 30 min prior to probing with primary and IR-dye conjugated secondary antibodies (LI-COR Biosciences, Lincoln, NE, USA) in PBS-T + 5% nonfat milk. Immunoblots were visualized using an Odyssey CLx infrared imaging system (LI-COR Biosciences). Quantification was performed using ImageStudio (LI-COR Biosciences).

### 2.6. Immunofluorescence Microscopy

U2OS cells were transfected with RepER plasmid using XtremeGeneHP (Roche). Cells were cultured in chamber slides (Millipore, Burlington, MA, USA) and infected with CVB (100 PFU/cell) for 7 h. Cells were fixed in 4% paraformaldehyde and permeabilized with 0.1% Triton X-100. Monolayers were washed, incubated with anti-VP1 primary antibody, washed, incubated with secondary antibody conjugated to Alexa Fluor-633 (Invitrogen), washed, and mounted with Vectashield containing 4′,6-diamidino-2-phenylindole (DAPI; Vector Laboratories, Burlingame, CA, USA). When indicated, samples were incubated with primary antibodies for 1 h at room temperature, washed, incubated with Alexa Fluor conjugated secondary antibodies for 30 min, and mounted using Vectashield (Vector Laboratories). Images were captured using a Zeiss LSM 710 confocal microscope (ZEISS, Oberkochen, Germany).

### 2.7. Long-Term Time-Lapse Fluorescent Live-Cell Imaging

U2OS cells stably expressing RepER or RepOr were plated into 35 mm dishes with a 14 mm glass no. 1.5 coverslip (MatTek Corporation, Ashland, MA, USA). Live-cell imaging was performed as previously described [33,34]. Briefly, dishes were placed in a 37 °C, CO_2_-controlled incubator positioned over a motorized, inverted fluorescent microscope to allow for long-term time-lapse imaging (VivaView FL; Olympus, Tokyo, Japan). Between 5 and 10 stage positions were selected for each sample. CVB (300 PFU/cell) was added to cells and images were captured every 15–20 min for 16–18 h. Image series were cropped using ImageJ software (NIH, Bethesda, MA, USA) and multi-panel movies were rendered using Photoshop CC 2017 (Adobe, San Jose, CA, USA).

### 2.8. Image and Data Analysis

#### 2.8.1. Intensity Profile Analysis and Nuclear Fluorescence Quantification of Confocal Images

The Plot Profile tool in ImageJ was used to perform line plot analyses on the red, green, and blue channels of confocal images. Data was plotted and the area under the curve was calculated for the area corresponding to the nucleus using Prism 8 software (GraphPad, San Diego, CA, USA).

ImageJ was used to determine the fluorescence intensity of the whole cell and nuclear, GFP and mCherry signals for individual cells. The cytoplasmic fluorescence intensity was calculated by subtracting the GFP value from the whole cell value. Due to variable fluorescence intensity between cells, we normalized nuclear fluorescence intensity to the cytoplasmic intensity. The ratio of GFP to mCherry was then calculated for each cell to determine if infection changed nuclear GFP fluorescence compared to mCherry.

#### 2.8.2. Infection Quantification

In order to account for changes in fluorescent signal throughout live-cell imaging, due to debris moving into the field of view, the fluorescent signal was normalized to an internal control. ImageJ was used to measure the mean signal intensities of a circular area within the nucleus and in ER-dense microtubule-organizing center region for individual cells from every image series frame. Infection was then calculated as the ratio between the nuclear and perinuclear signals. Nonlinear regression curve analysis in Prism 8 (Graphpad) was used to determine the time at which 50% of the maximum infection signal was observed for individual infected cells.

#### 2.8.3. ER Integrity Quantification

Peripheral ER integrity was determined by quantifying the ratio of peripheral ER signal to dense ER sheet-like signal. Images were thresholded to exclude the fine ER structures in the cell periphery. The area of the nucleus was subtracted from the area of the thresholded region to calculate the ER sheet area. Next, a boundary was drawn around the perimeter of the ER network and the area was measured. The areas of the nucleus and ER sheets were subtracted from this value to calculate the peripheral ER area. The ratio of peripheral ER to ER sheet area was used to quantify peripheral ER integrity. These measurements were performed on individual cells from every image series frame using the same thresholding values.

#### 2.8.4. Golgi Area Quantification

Golgi dispersal was measured by quantifying Golgi area, which was performed similarly to previously described methods for imaging flow cytometry [35]. The first frame of an image series was thresholded to include the high-intensity cluster of mEmerald signal, indicative of the Golgi, for a single cell, and this area was measured. This measurement was taken for the same cell for every frame of an image series using the same thresholding value. This was performed for individual cells from every image series frame.

#### 2.8.5. Effects of 2APB Treatment

Image series were used to determine the percentage of infected cells at 8 hpi by observing infection reporter translocation. Infected cells with aberrant ER structures at 8 hpi in the image series were manually counted using ImageJ (NIH). Image series were used to calculate the percentage of lysed cells, which was determined by counting the number of cells showing infection at 8 hpi, then tracking these cells up to 16 hpi and looking for the loss of the cytoplasmic reporter signal due to loss of plasma membrane integrity.

#### 2.8.6. Statistics

Student’s *t*-tests and one-way ANOVA with Bonferroni multiple comparisons tests were performed using Prism 8 software (Graphpad).

## 3. Results

### 3.1. Enterovirus Reporter Plasmid Construction.

**Figure 1:**
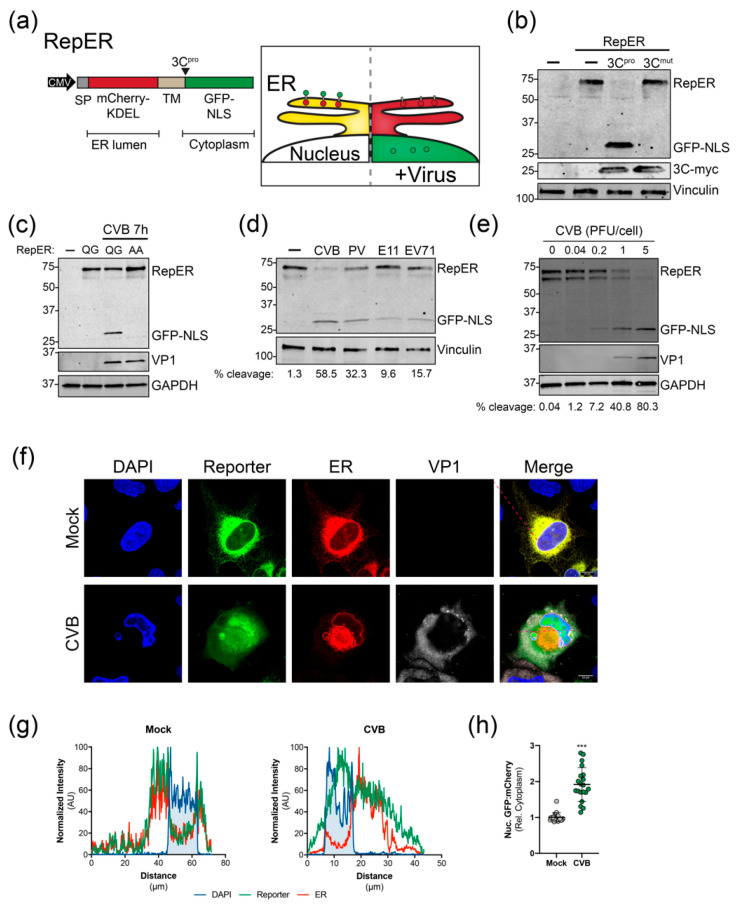
Validation of enterovirus plasmid-based reporters. (**a**) *Left*, RepER cassette driven by the CMV promoter. The RepER cassette contains an ER-localized mCherry containing a signal peptide (SP) and KDEL ER retention sequence followed by signal peptidase site fused to a transmembrane domain (TM) followed by the viral 3C protease (3C^pro^) recognition sequence and GFP fused to a nuclear localization sequence (NLS). *Right*, Schematic of the predicted model for visualizing the ER and enterovirus infection. In uninfected cells (left) mCherry and GFP colocalize in the ER. During enterovirus infection (right,) viral 3C^pro^ cleavage releases the GFP-NLS, which translocates to the nucleus. (**b**) Immunoblot of GFP, myc, and vinculin from lysates from U2OS cells transfected with RepER and empty (-), CVB 3C^pro^-myc, or catalytically inactive CVB 3C^pro^-myc (3C^mut^) expression plasmids. (**c**) Immunoblot for GFP, VP1, and GAPDH from lysates from CVB infected (100 PFU/cell) U2OS cells transfected with RepER or RepER containing an AA mutation to the QG in the 3C^pro^ recognition sequence. The presence of a weak lower RepER band indicates poor host signal peptidase-dependent release of mCherry-KDEL from the TM anchor. (**d**) Immoblot of GFP and vinculin from lysates from HeLa cells transfected with RepER followed by infection (1 PFU/cell) with CVB, poliovirus (PV), echovirus 11 (E11), or enterovirus 71 (EV71) for 7 h. Quantification of percent cleavge is shown below. (**e**) Immoblot for GFP, VP1, and GAPDH of HeLa cells expressing RepER infected with CVB at the indicated PFU/cell for 7 h. Quantification of percent cleavge is shown below. (**f**) Representative confocal images of mock and CVB infected U2OS cells expressing RepER stained for DAPI (blue) and CVB VP1 (gray), scale bars are 10 μm. (**g**) Normalized signal intensities of DAPI, GFP (reporter), and mCherry (ER) from line plot analyses of confocal images shown in (**f**), which show higher GFP signal in the DAPI region upon CVB infection. (**h**) Quantification of the nuclear fluorescence intensity ratio of GFP to mCherry relative to the cytoplasmic fluorescence intensity of an internal standard for each cell (*n* = 20). Significance was determined by Student’s *t*-test, *** *p* < 0.0001.

To monitor enterovirus infection in real-time, we adapted cell-based reporter methodologies previously used for flaviviruses and hepatitis C virus that rely on viral protease cleavage-dependent translocation of a membrane-anchored cytoplasmic fluorescent proteins to the nucleus [27,28,29]. However, given that these systems do not allow for the visualization of virus-induced manipulation of host cell organelles, we sought to design reporter plasmids expressing a fluorescent protein to indicate infection in addition to fluorescent protein-targeted organelle markers. As a proof of principle, we developed a dual reporter construct (RepER) that expresses an ER lumen localized mCherry containing an ER retention signal (KDEL) and signal peptidase cleavage site fused to a type I transmembrane domain followed by an enterovirus 3C protease (3C^pro^) target sequence and a cytoplasmic GFP containing a nuclear localization signal (GFP-NLS) (Figure 1a). Upon infection, the expression of 3C^pro^ is predicted to release the ER-tethered GFP-NLS, allowing for translocation to the nucleus (Figure 1a). Importantly, given that the mCherry-KDEL remains localized in the ER, we can monitor ER dynamics in parallel.

We first validated the cleavage of the reporter by expressing myc-tagged CVB 3C^pro^ and 3C^mut^, a catalytically inactive protease mutant, in U2OS cells. Immunoblot analysis of cell lysates indicated that expression of CVB 3C^pro^ led to the efficient release of GFP-NLS from the full RepER protein, whereas cleavage was absent in 3C^mut^ expressing cells (Figure 1b). To ensure that cleavage of RepER is dependent on viral protease cleavage during infection, we generated a construct containing a double alanine mutation (AA) of the glutamine-glycine (QG) 3C^pro^ cleavage site. We found that mutation of the viral protease cleavage site blocked CVB-induced cleavage of RepER (Figure 1c). Next, we sought to determine if RepER was cleaved by a panel of enteroviruses during infection. We found that infection of RepER-expressing HeLa cells with CVB, poliovirus, echovirus 11, and enterovirus 71 resulted in cleavage of RepER to varying degrees (Figure 1d) and efficiency of cleavage correlated with MOI (Figure 1e). Lastly, using immunofluorescence microscopy, we verified that CVB infection resulted in translocation of GFP-NLS to the nucleus, whereas mCherry was retained in the ER (Figure 1f). Notably, we did not observe complete GFP-NLS translocation, which is consistent with previous reports demonstrating that enterovirus infection restricts nuclear transport [36]; additionally, there may be uncleaved reporter present on the ER, which can account for some of the cytoplasmic GFP fluorescence. Intensity profile analysis of confocal images showed that mock-infected cells had lower GFP-NLS reporter and mCherry-ER signal intensities in the nuclear region, defined by peaks of DAPI signal (Figure 1g). Importantly, CVB infection led to an increase in GFP-NLS reporter signal intensity in the nuclear region, while mCherry-ER maintained a lower signal intensity in this region. Upon quantification of confocal images, we observed a significant increase in the ratio of GFP to mCherry signals in the nucleus relative to the cytoplasmic during CVB infection compared to mock (Figure 1h). Together, these results indicate that the RepER reporter can be used to efficiently detect infection by a broad range of enteroviruses and monitor virus-induced changes of the ER.

### 3.2. Live-Cell Imaging Demonstrates Virus-Induced Manipulation of the ER

**Figure 2:**
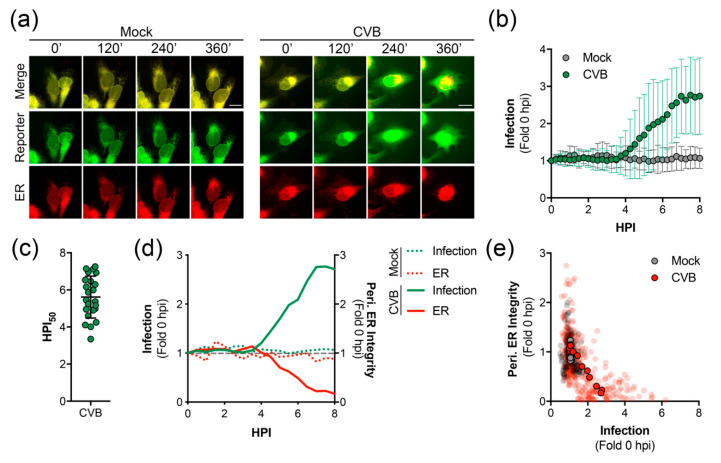
Time-lapse imaging of CVB infected cells expressing RepER. (**a**) Representative time-points of an image series from live-cell imaging of GFP (reporter, green), mCherry (ER, red), and merged panels from mock or CVB infected U2OS cells expressing RepER. Scale bars are 20 μm. (**b**) Quantification of infection in individual cells, defined as the ratio of nuclear to ER-dense microtubule-organizing center signal intensities, from individual RepER expressing cells either mock (*n* = 9) or CVB (*n* = 23) infected. Measurements were taken every 15 min, and data is shown as the average ± SD of the fold change in infection signal compared to 0 hpi. (**c**) Determination of the time point at which 50% maximum infection signal was observed (HPI_50_) as determined by nonlinear regression curve analysis for each CVB infected cell. Each data point shown is the HPI_50_ for an individual cell (*n* = 23), and the line represents the average ± SD. (**d**) Quantification of infection (green) and peripheral ER integrity (red) for the mock (dashed lines, *n* = 9) and CVB (solid lines, *n* = 23) infected cells shown as the average fold change compared to 0 hpi; the gray dashed line shows fold change of 1. (**e**) Scatter plot of infection and peripheral ER integrity. Transparent dots represent individual measurements taken every 15 min for individual mock (gray, *n* = 9) or CVB (red, *n* = 23) infected cells from 0 to 8 hpi; solid outlined dots represent the averages.

We next utilized long-term time-lapse imaging to determine the kinetics of GFP-NLS translocation during virus infection. We used U2OS cells, which are highly conducive to live-cell imaging. CVB infection of RepER expressing U2OS cells resulted in a clear translocation of GFP-NLS reporter to the nucleus and eventual dispersal due to cell lysis, while the mCherry-KDEL was maintained in the membranous structure of the ER (Video S1 and Figure 2a). Image series analysis revealed that infected cells exhibited a linear increase in nuclear translocation of GFP-NLS starting around ~4 hpi on average (Figure 2b) with 50% of the maximum infection signal occurring ~5.5 hpi (Figure 2c), whereas mock-infected cells showed no increase in nuclear GFP signal.

The ER has recently been shown to be involved in the early stages of CVB RO formation [15]. Thus, we sought to determine virus-induced changes to the ER throughout infection using long-term time-lapse imaging. We observed a dramatic loss of the structural integrity of the ER, as shown by the collapse of the peripheral ER network in cells infected with CVB (Video S1, Video S2, and Figure 2a). Analysis of image series from individual cells indicated a decrease in the ratio of peripheral ER to sheet areas, a measurement of peripheral ER integrity, that coincided with an increase in signal for infection (Figure 2d). Furthermore, analysis of multiple image series showed a reverse correlation between the infection signal and the peripheral ER integrity (Figure 2e). Interestingly, we found that the collapse of the peripheral ER network preceded cell lysis by ~7 h (Videos S1 and S2).

### 3.3. Live-Cell Imaging Demonstrates Virus-Induced Golgi Dispersal

**Figure 3:**
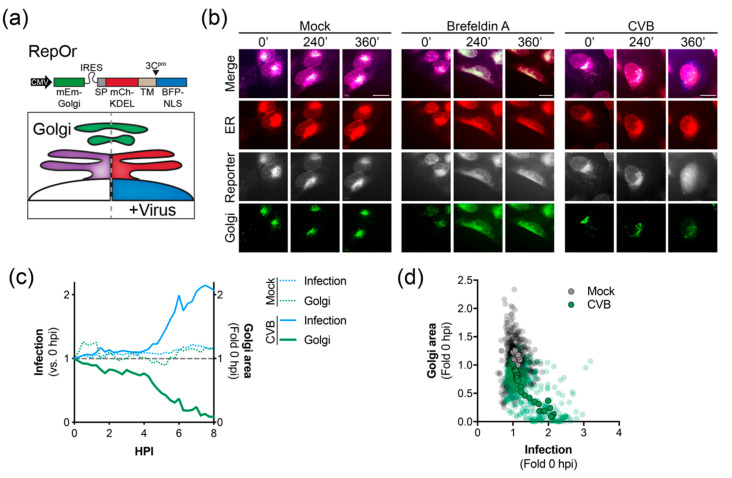
Time-lapse imaging of CVB infected cells expressing RepOr. (**a**) *Top*, the bicistronic RepOr cassette contains a Golgi localized mEmerald (mEm-Golgi) followed by an IRES-driven RepER, where the GFP has been substituted with BFP. Signal peptide (SP), transmembrane domain (TM), and nuclear localization signal (NLS). *Bottom*, Schematic of the predicted model for visualizing the Golgi, ER, and enterovirus infection. In uninfected cells (left), mCherry and BFP colocalize in the ER (purple), and mEmerald is localized to the Golgi (green). During enterovirus infection (right), viral 3C^pro^ cleavage releases the BFP-NLS, which translocates to the nucleus, the mCherry is retained in the ER (red) and the Golgi remains (green). (**b**) Representative time-points of an image series from live-cell imaging of mEmerald (Golgi, green), BFP (reporter, gray), mCherry (ER, red), and merged panels from U2OS cells expressing RepOr treated with DMSO, brefeldin A, or infected with CVB. Scale bars are 20 μm. (**c**) Quantification of infection (blue) and Golgi area (green) in mock (dashed lines, *n* = 16) and CVB (solid lines, *n* = 16) infected cells shown as the average fold change compared to 0 hpi; the gray dashed line shows a fold change of 1. (**d**) Scatter plot of infection and Golgi areas. Transparent dots represent individual measurements taken every 15 min for individual mock (gray, *n* = 16) or CVB (green, *n* = 16) infected cells from 0 to 6 hpi, solid outlined dots represent the averages.

Enterovirus infection has been previously shown to restrict secretory trafficking, resulting in Golgi dispersal [14,16,17,18,19,20]. Thus, we developed a bicistronic, triple reporter to visualize the ER and Golgi as well as monitoring virus infection (RepOr). The RepOr construct encodes an mEmerald-Golgi marker followed by an IRES-driven RepER cassette that has been modified to include a BFP-NLS reporter protein (Figure 3a). Time-lapse imaging of cells expressing RepOr showed correct localization of organelle reporters that were maintained throughout time-lapse imaging (Video S3 and Figure 3b). To initially validate this construct, we performed time-lapse imaging of RepOr cells treated with brefeldin A (BFA), an inhibitor of ER to Golgi protein transport [37]. As expected, BFA treatment resulted in a clear change in the localization of the Golgi marker from a tight cluster to a dispersed signal that colocalized with the ER marker, which indicated a disruption of Golgi integrity and a block in protein transport out of the ER (Video S4 and Figure 3b). Next, we sought to monitor virus-induced changes to the Golgi during infection of RepOr expressing cells. To do this, we performed time-lapse imaging of cells infected with CVB. Consistent with previous reports [14,16,17,18,19,20,38], CVB infection led to a clear dispersal of the Golgi marker, as shown by a decrease in the high-intensity Golgi signal area and presence of a diffuse low-intensity signal, indicating a disruption of Golgi integrity (Video S5 and Figure 3b). Analysis of this image series indicated that dispersal of the Golgi coincided with an increase in the signal for infection (Figure 3c) and analysis of multiple image series showed a reverse correlation between infection signal and Golgi area (Figure 3d). Together, these results indicated the RepOr construct can be used to monitor pharmacological and viral manipulation of the secretory pathway.

### 3.4. Reporter Constructs Do Not Restrict Replication Kinetics

**Figure 4:**
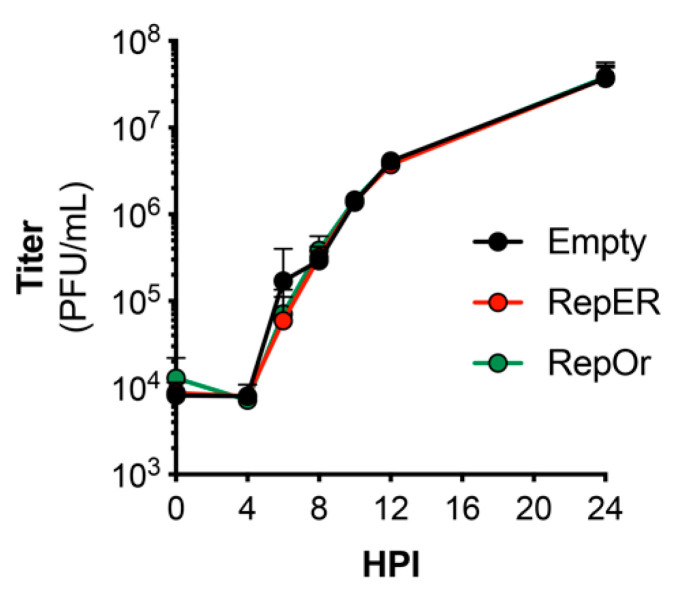
Growth kinetics of CVB in reporter expressing cells. U2OS cells transfected with the indicated expression plasmids were bound with CVB (100 PFU/cell) at 4 °C for 1 h, washed, and infection was allowed to proceed at 37 °C for 24 h. Plaque assays were performed on samples collected at the indicated time points. Data represents the average ± SD titer (plaque-forming units [PFU]/mL) from two independent experiments.

Previous studies have utilized recombinant enteroviruses that encode fluorescent protein reporters to visualize infection in real-time. However, these viruses display dramatic defects in replication kinetics [14,25,26]. Thus, we sought to determine if reporter plasmid expression attenuates virus replication. We performed single-step growth curves for CVB in U2OS cells transfected with each reporter plasmid. Our results indicated there were no differences in growth of CVB in U2OS cells transfected with empty, RepER, or RepOr plasmids (Figure 4). These results suggest our reporter plasmids can be used to accurately monitor viral replication kinetics and virus-induced manipulation of host cells in real-time.

### 3.5. CVB-Induced ER Reorganization Is Altered by Calcium Channel Inhibition

**Figure 5:**
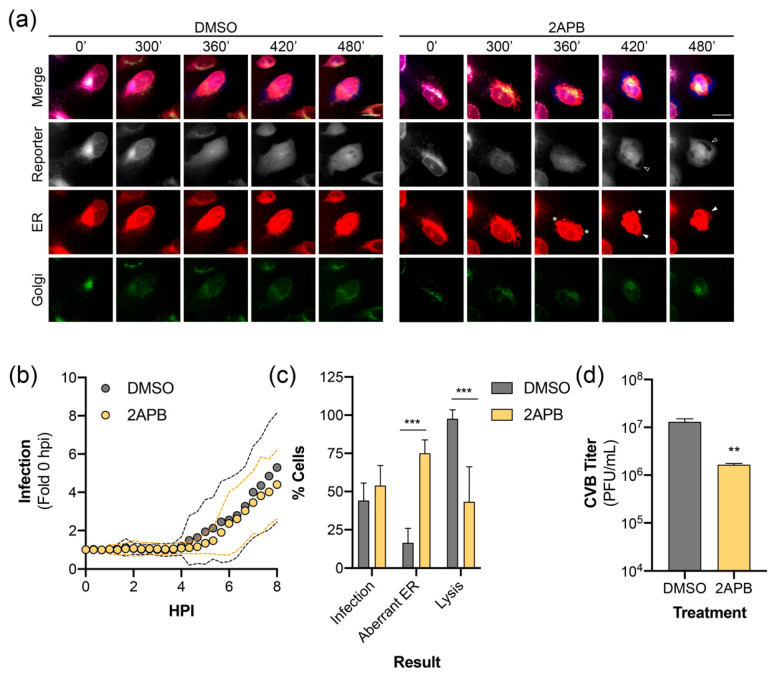
ER calcium channel inhibition alters CVB-induced ER remodeling. (**a**) Representative time points of an image series from live-cell imaging of mEmerald (Golgi, green), BFP (reporter, gray), mCherry (ER, red), and merged panels from U2OS cells expressing RepOr treated with DMSO or 2APB (100 μM) with CVB. Aberrant ER structures are indicated as protein aggregates (*), inflated ER (white arrowhead in ER panels), which exclude cytoplasmic BFP (empty arrowheads in reporter panels). Scale bars are 20 μm. (**b**) CVB infection kinetics of individual U2OS cells expressing RepOr treated with DMSO (*n* = 24) or 2APB (100 μM, *n* = 24). Data is shown as the average (dots) ± SD (dashed lines) infection signal every 20 min from 0 to 8 hpi, presented as fold change compared to 0 hpi. (**c**) Phenotypes of cells from live-cell imaging of DMSO or 2APB (100 μM) treated U2OS cells expressing RepOr infected with CVB. Data is shown as the percentage of cells showing nuclear translocation of the reporter at 8 hpi (Infection), percentage of cells showing infection containing aberrant ER structures (Aberrant ER), or the percentage of cells showing reporter translocation at 8 hpi that resulted in lysis by 16 hpi (Lysis), as determined by loss of reporter signal upon loss of membrane integrity (as described in Materials and Methods 2.8.5). The data was collected from two independent experiments, with four separate fields/experiment. Significance was determined by one-way ANOVA, *** *p* < 0.0005. (**d**) Titer of extracellular virus from CVB infected U2OS cells treated with DMSO or 2APB (100 μM) at 10 hpi. The data is shown as the average ± SD titer (PFU/mL) from three independent experiments. Significance was determined by student’s *t*-test, ** *p* < 0.005.

Lastly, we sought to demonstrate the utility of our reporter system by visualizing the effect of a pharmacological inhibitor on virus infection. We have previously reported that inhibition of inositol 1,4,5-triphosphate receptors (IP_3_R), which function as calcium channels in the ER, restricts the release of an infectious virus through modulating cell death pathways [39]. Long-term time-lapse imaging of CVB infected RepOr cells treated with DMSO produced results similar to those described above (Video S6, Video S7, and Figure 5a). Time-lapse imaging of RepOr cells in the presence of 2-aminoethoxydiphenylborane (2APB), an IP_3_R inhibitor, did not prevent the nuclear translocation of the virus reporter or dispersal of the Golgi (Video S8, Video S9, and Figure 5a). Furthermore, image series analysis showed no defect in the kinetics of nuclear translocation of the reporter, indicating this drug does not affect viral protein production and replication (Figure 5b). Additionally, we did not observe a difference in the percentage of cells infected at 8 hpi, indicated by reporter translocation to the nucleus (Figure 5c). Interestingly, 2APB treatment of cells led to the formation of aberrant ER structures that coincided with reporter translocation to the nucleus (Video S8 and Video S9). These structures resembled large membrane aggregates and inflated regions of ER (Figure 5a, asterisks and arrowheads, respectively), which were rarely observed in infected DMSO-treated cells (Figure 5c) and not observed in uninfected cells. Furthermore, treatment with 2APB led to a significant decrease in the number of infected cells that progressed to lysis (Figure 5c), which is consistent with a significant decrease in the titer of extracellular virus (Figure 5d). Together, these results indicate that our plasmid-based reporters can be used to monitor virus-induced changes to host membranes upon inhibition of specific cellular functions, which can further our understanding of inhibitor mechanisms of action during infection.

## 4. Discussion

Live-cell imaging is a powerful tool to study virus-host interactions in real-time. To facilitate this, we developed and characterized multipartite fluorescent-reporters to monitor enterovirus-induced remodeling of organelles in the host secretory pathway. These reporters rely on enterovirus 3C^pro^ activity to release an NLS-tagged fluorescent protein, which translocates to the nucleus while retaining ER and Golgi localized fluorescent markers. Live-cell imaging of CVB infected cells expressing these reporters allowed for the real-time visualization of virus-induced changes to the host cell, including the collapse of the peripheral ER network and loss of Golgi integrity. Furthermore, we used live-cell imaging with RepOr to show that a calcium channel inhibitor, 2APB, resulted in CVB-induced formation of aberrant ER structures and decreased the lytic effects of the virus. Overall, we show that our reporters present innovative opportunities for studying enterovirus-induced remodeling of host cell membranes during infection in real-time.

Previous studies have utilized plasmid-based reporters for live-cell imaging of other small positive-strand RNA virus infections [27,28,29]. Plasmid-based reporters for dengue virus and Zika virus utilized viral nonstructural protein 4B (NS4B), a multi-pass ER membrane-anchored protein, fused to GFP-NLS [28,29]. However, expression of flavivirus NS4B has previously been associated with the induction of autophagy and remodeling of the ER, which can complicate interpretation of results and restrict the applications in which these reporters can be used [40,41]. Additionally, these reporters are limited to the detection of virus infection. To monitor virus-induced changes to the host-cell they would need to be used in combination with other fluorescent-marker expression vectors or stable cells. Thus, we expanded on these reporters to develop multipartite fluorescent reporters that anchor the virus reporter via a single-pass host-derived transmembrane domain. This design eliminates the need for transfection of multiple constructs to visualize host structures and virus infection and significantly simplifies the generation of stable cells or transgenic animals. Whereas the flavivirus reporters are virus-specific [28], the RepER and RepOr reporters can be used for a broad array of enteroviruses. While all enterovirus infections tested resulted in reporter cleavage, there were differences in efficiency with CVB infection consistently resulting in the highest reporter cleavage efficiency. One explanation is the engineered cleavage site may not be optimal for all enteroviruses and may need to be modified for specific viruses. Alternatively, there may be slight differences in replication kinetics between these viruses, which results in lower levels of cleavage by the time point tested. Thus, these reporters can be used to compare virus-specific manipulation of host membranes in a wide variety of target cells.

All positive-strand RNA viruses remodel host membranes to form ROs that facilitate the sequestration of viral and host factors to promote efficient replication [7,8,9,10]. Recently, a recombinant CVB was developed to show in real-time that the accumulation of the viral 3A coincides with Golgi disruption [14]. This recombinant virus was also used to identify the ER as an early source for RO membranes [15]. However, the manipulation of the CVB genome to introduce fluorescent-based reporters resulted in dramatic attenuation, which is common in the development of most recombinant small positive-strand RNA viruses [14,25,26]. An important benefit of our reporters is their expression did not restrict CVB infection. Thus, we utilized the reporters to evaluate the kinetics of CVB-induced membrane remodeling at a single-cell level. Using live-cell imaging and image series analysis, we were able to demonstrate that Golgi dispersal and loss of peripheral ER integrity coincided with an increase in CVB infection. Interestingly, the diffuse reporter signal in the cytoplasm indicated that the loss of the peripheral ER network did not coincide with cell rounding, shrinkage, or result in a sudden progression to cell death, which occurred ~7 h post ER collapse. Interestingly, CMV infection leads to the collapse of the ER due to the expression of the viral pUL37×1 protein [42]. Thus, future studies are warranted to determine if interactions between viral and host proteins are responsible for CVB-induced remodeling of the ER.

We applied our reporter systems to better understand the effects of calcium channel inhibition on infection, which we have previously shown decreases the release of infectious virus [39,43]. We found that 2APB inhibition of calcium channels did not block virus-induced nuclear translocation of the virus reporter, Golgi dispersal, or ER collapse. However, we did find that 2APB treated cells showing virus reporter translocation to the nucleus contained aberrant ER structures and prevented cell lysis. Previous studies have shown similar structures represent ER whorls or stacked ER, which have been proposed to regulate cargo exit from the ER [44,45,46]. In yeast, these structures have also been associated with induction of ER stress and the unfolded protein response [47,48], which could explain their presence in CVB infected cells that are unable to regulate Ca^2+^ release. Thus, our results demonstrate an important application for our reporters in understanding the real-time effects of pharmacological inhibitors on virus-induced manipulation of the host cell.

Overall, we have shown the benefits of using multipartite plasmid-based reporters to visualize virus-induced remodeling of the host cell using live-cell imaging. Our system can be readily modified to include combinations of other fluorescent markers of host cell structures or viral host factors. Additionally, these constructs can be modified to include recognition sites for proteases encoded by other positive-strand RNA viruses, including flaviviruses, alphaviruses, and coronaviruses. Furthermore, these reporters can be used for other applications, including diagnostics and high-throughput screens.

## Supplementary Materials

The following are available online at https://www.mdpi.com/1999-4915/12/10/1074/s1, Table S1: Primer and oligonucleotide sequences, Video S1: Real-time imaging of CVB infected RepER expressing cells, Video S2: Real-time imaging of an individual CVB infected RepER expressing cell, Video S3: Real-time imaging of DMSO treated RepOr cells, Video S4: Real-time imaging of RepOr cells treated with brefeldin A, Video S5: Real-time imaging of RepOr cells infected with CVB, Video S6: Real-time imaging of DMSO treated RepOr cell monolayer, Video S7: Real-time imaging of an individual DMSO treated RepOr cell, Video S8: Real-time imaging of 2APB treated RepOr cell monolayer, Video S9: Real-time imaging of an individual 2APB treated RepOr cell. Supplemental File Legends.
