# Supplementary material for: Imaging-Based Reporter Systems to Define CVB-Induced Membrane Remodeling in Living Cells
Source: Viruses. 2020 Sep 25;12(10):1074. doi: 10.3390/v12101074 (PMC7600424; doi:10.3390/v12101074)
Supplement: Supplementary file 1 [file viruses-12-01074-s001.zip › Supplemental/Supplemental Legends.docx]

**VIDEO S1** Real-time imaging of CVB infected RepER expressing cells. U2OS cells expressing RepER infected with mock or CVB (300 PFU/cell) were imaged every 15 minutes for 18 h. Shown are ER (red), reporter (green), and merged panels from a representative field.

**VIDEO S2** Real-time imaging of a CVB infected RepER expressing cell. U2OS cells expressing RepER infected with mock or CVB (300 PFU/cell) were imaged every 15 minutes for 18 h. Shown are zoomed-in reporter (green), ER (red), and merged movies from a representative cell from Video S1.

**VIDEO S3** Real-time imaging of DMSO treated RepOr cells. U2OS cell expressing RepOr were treated with DMSO and imaged every 15 minutes for 18 h. Shown are merged, Golgi (green), reporter (gray), and ER (red) movies of representative cells.

**VIDEO S4** Real-time imaging of RepOr cells treated with brefeldin A. U2OS cell expressing RepOr were treated with brefeldin A (5 μg/mL) and imaged every 15 minutes for 18 h. Shown are merged, Golgi (green), reporter (gray), and ER (red) movies of representative cells.

**VIDEO S5** Real-time imaging of RepOr cells infected with CVB. U2OS cells expressing RepOr were infected with CVB (300 PFU/cell) and imaged every 15 minutes for 18 h. Shown are merged, Golgi (green), reporter (gray), and ER (red) movies from a representative cell.

**VIDEO S6** Real-time imaging of DMSO treated RepOr cells. U2OS cell expressing RepOr were treated with DMSO, infected with CVB (300 PFU/cell), and imaged every 20 minutes for 16 h. Shown are merged, Golgi (green), ER (red), and reporter (gray) movies of a representative field of cells.

**VIDEO S7** Real-time imaging of DMSO treated RepOr cells. U2OS cell expressing RepOr were treated with DMSO, infected with CVB (300 PFU/cell), and imaged every 20 minutes for 16 h. Shown are merged, Golgi (green), ER (red), and reporter (gray) movies of a representative cell from Video S6.

**VIDEO S8** Real-time imaging of 2APB treated RepOr cells. U2OS cell expressing RepOr were treated with 2APB (100 μM), infected with CVB (300 PFU/cell), and imaged every 20 minutes for 16 h. Shown are merged, Golgi (green), ER (red), and reporter (gray) movies of a representative field of cells.

**VIDEO S9** Real-time imaging of 2APB treated RepOr cells. U2OS cell expressing RepOr were treated with 2APB (100 μM), infected with CVB (300 PFU/cell), and imaged every 20 minutes for 16 h. Shown are merged, Golgi (green), ER (red), and reporter (gray) movies of a representative cell from Video S8.
